# Accuracy of second and third trimester ultrasound in the determination of chorionicity in twin pregnancies: a cross-sectional observational study

**DOI:** 10.1186/s12884-025-07404-7

**Published:** 2025-06-02

**Authors:** Chantal Stewart, Zoe Momberg, Henriette van Zyl, Stephen Lindow

**Affiliations:** 1 Department of Obstetrics & Gynaecology, Groote Schuur Hospital, University of Cape Town, H-Floor, Old Main Building, Anzio Road, Observatory, Cape Town, South Africa; 2 Director of Masters Projects, The Coombe Hospital, Dublin, Ireland

**Keywords:** Twin chorionicity, Membrane thickness, Layers in membrane, Lambda sign, Discordant sexes, Number of placentae

## Abstract

**Background:**

Determination of chorionicity in twin pregnancies is essential for optimizing management. In South Africa, many women first access antenatal care at late gestations. The accuracy of ultrasound in the determination of chorionicity up to 14 weeks’ gestation is well documented. However, there is little data on determination of chorionicity in late pregnancy. We thus aimed to determine the accuracy of five ultrasound parameters performed after 14 weeks’ gestation in determining chorionicity.

**Method:**

This was a cross-sectional diagnostic validity study where postpartum placental histology was accepted as the standard test for chorionicity. All twin pregnancies > 14 weeks’ gestation in whom chorionicity had not been determined and without any complications were included. Data collected included demographic data, ultrasound-determined number of placentae, fetal sexes, membrane ‘take-off’ (lambda or T-Sign), thickness, and number of layers in the dividing membrane. Placentae and membranes were examined by the Anatomical Pathology department. Each of the 5 ultrasound parameters for chorionicity was analyzed separately in relation to the corresponding histology result.

**Results:**

A total of 85 patients were included, for whom histology was available in 58. Of these, 12 were monochorionic diamniotic and 44 dichorionic diamniotic. The diagnostic accuracies of discordant gender, number of placentas, lambda sign, membrane thickness and number of layers in the membranes were 44.1, 49.1, 76.7, 76.1 and 82.9, respectively. We developed a step-wise algorhithm for the determination of chorionicity.

**Conclusion:**

Determination of chorionicity late in twin pregnancies remains inaccurate. However, a step-wise algorithm for the application of 5 parameters is helpful.

## Synopsis

Determination of chorionicity at late gestations in twin pregnancies may be improved by combining five ultrasound parameters in a step-wise algorithm.

## Introduction

Twin pregnancies are monochorionic (MC) in 20% of cases and these have a relative risk of 2.5 times that of dichorionic (DC) twins for perinatal mortality of at least one twin [1, 2]. In addition, there is a higher incidence of fetal complications in MC versus DC pregnancies. These include preterm labor, intrauterine growth restriction (IUGR), fetal distress, and delivery by cesarean Sects. [3, 4] There are also complications specific to MC pregnancies, such as twin- to-twin transfusion syndrome (TTTS), twin reversed arterial perfusion (TRAP) sequence, selective IUGR, cord entanglement, and conjoined twins [1, 5, 6].

Management differs between MC and DC twins in terms of the frequency of antenatal visits and timing of delivery [7, 8]. In addition, complications such as a fetal abnormality in one of a twin pair or a single intrauterine fetal death will have different prognoses and will require different forms of management depending on the chorionicity [9]. Thus chorionicity plays a crucial role in informing management decisions and predicting outcomes.

First trimester sonography, using identification of the lambda or T-signs, has been proven to be very accurate in determining chorionicity, with reported sensitivities and specificities of 100% and 99%, respectively, if performed < 14 weeks’ gestation [10–12]. After 14 weeks’ gestation, the accuracy of ultrasound is thought to decline, as identification of the lambda sign becomes more difficult and will disappear by the 20th week of pregnancy in 7% of DC pregnancies [13].

Other parameters used to determine chorionicity have included number of placentae [14] and fetal sexes [11]. Measurement of the inter-twin membrane thickness and counting the number of membrane layers have been reported as less reliable than the lambda or T-sign [15]. Using a cut-off membrane thickness of 2 mm, accuracies ranging from 52 to 96.6% have been reported [11, 16–18]. Counting of the membrane layers (four layers indicating a DC pregnancy and two layers an MC pregnancy) has a reported positive predictive value (PPV) of 98,5% and an accuracy approaching 100% in DC gestations [11]. A review by Shetty quoted accuracy rates of 97% in the third trimester if a composite cascade of ultrasound features was used to determine chorionicity [15].

Perinatal mortality is higher in South Africa than in high income countries and some of this is related to late presentation for antenatal care. In twin pregnancies, late pregnancy determination of chronicity is common in South Africa, as only 21% of women initiate antenatal care before 20 weeks’ gestation [19].

We thus aimed to determine the accuracy in determining chorionicity of three parameters that are commonly used: fetal sexes, number of placental masses and lambda sign after 14 weeks’ gestation. We further aimed to evaluate whether the additional signs of membrane thickness and number of membrane layers led to a significant improvement in accuracy.

## Methods

### Study design

This was a cross-sectional, diagnostic validity study where postpartum histology (macroscopic and microscopic) of placentae was accepted as the standard test for chorionicity [20, 21].

### Patient sample

Patients with twin pregnancies at gestations > 14 weeks attending antenatal clinics at a tertiary or secondary hospital in the Western Cape Province of South Africa between 1 February and 31 August 2011 were included. Exclusion criteria were.

(1) chorionicity previously determined by ultrasound ≤ 14 weeks, (2) monoamniotic twins, (3) pre-existing TTTS, (4) preterm premature membrane rupture (5) patient objection to placental histology and 5) woman < 18 years of age.

### Data analysis

Data collected included demographic data, ultrasound-determined number of placentae, fetal sexes, membrane ‘take-off’ (lambda or T-Sign), thickness, and number of layers in the dividing membrane.

The following criteria were used to classify pregnancies as DC: two distinct placentae at different locations, discordant fetal sex, presence of lambda sign, dividing membrane containing 3 or 4 layers, dividing membrane thickness > 2 mm.

If it was not possible to obtain all five parameters at the initial scanning visit, this was attempted at the next routine twin scan. A senior sonographer who had read the literature regarding the technique of measurement of the twin membrane thickness and number of layers in the membrane gave hands-on tuition to the sonographers who were less familiar. Emphasis was placed on the magnification of the image and having the membrane horizontal on the screen. Sonographers were observed for 5 cases each after the training and were encouraged to discuss their images with senior sonographers if there were doubts.

After delivery, placentae and membranes were examined by the Anatomical Pathology Department. If the chorionicity was clear macroscopically (intervening membrane seen between two separate placentae), then this was the final and conclusive test. If, however, it was difficult to determine macroscopically whether two placentae were closely adjacent with no anastomoses or if there was a single placental mass, microscopic histology of the dividing membrane was performed.

### Statistical analysis

A contingency table was formulated. STATA software ver 13 (StataCorp LLC, Texas, USA) was used to perform the analysis. The respective sensitivity, specificity, positive predictive value (PPV) and negative predictive value (NPV) were calculated for each parameter. A value of *p* < 0.05 was used to indicate statistical significance.

### Ethics

Informed consent was obtained from each patient and ethics approval was obtained from the University of Cape Town Health Research Ethics Committee (number HREC234/2011).

## Results

**Fig. 1:**
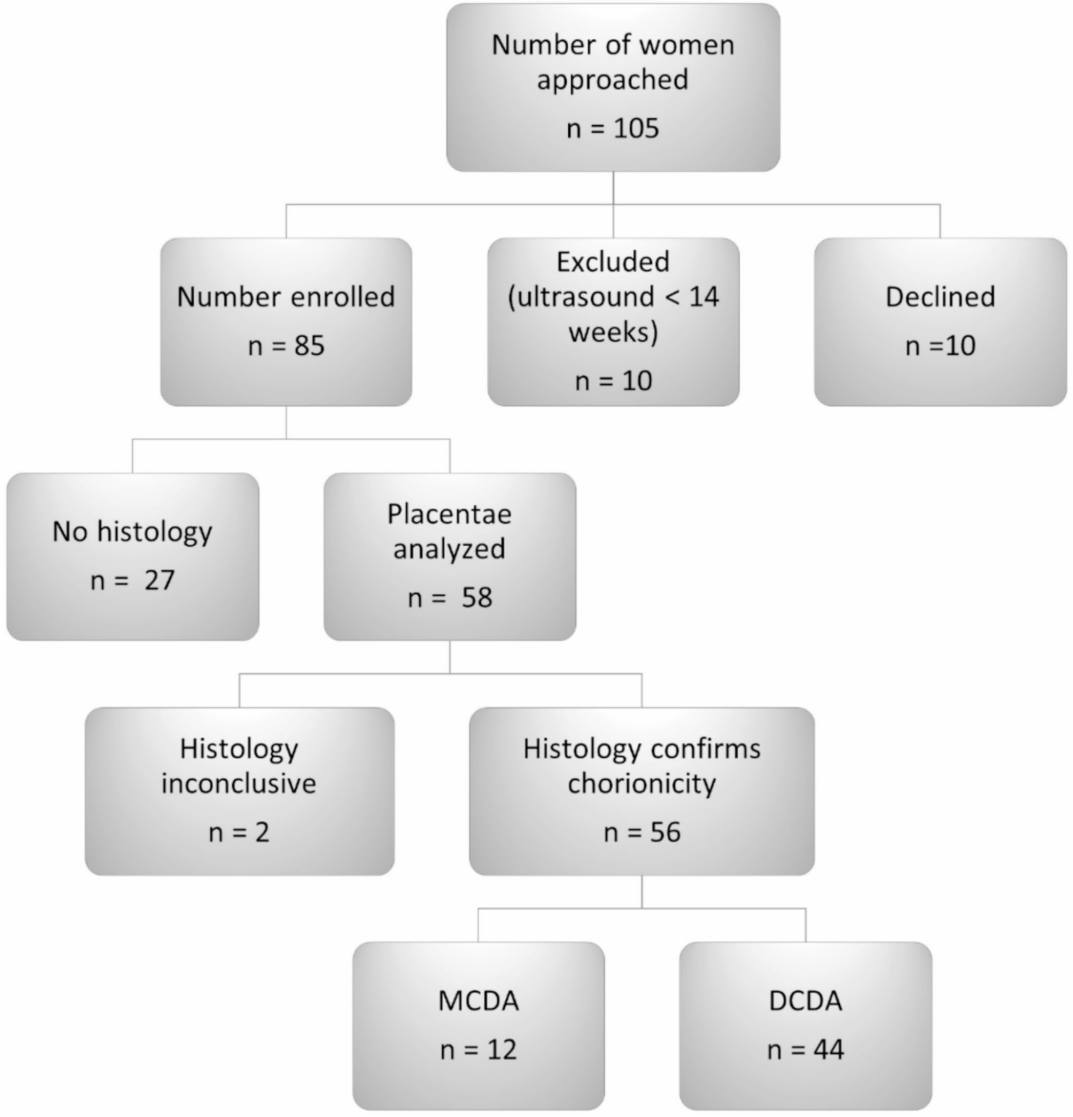
Data flow chart

Figure 1 outlines the data flow. The gestational age range at first scan was 15–31 weeks with eight patients having their first scan in the third trimester and the remainder in the second trimester.

Of the 85 patients enrolled, 58 sets of placentae were available for histological examination. Two placental sets had inconclusive histology due to incorrect site of membrane sampling or lack of identification of an area of fused membranes. The remaining 56 histology results were compared with the ultrasound characteristics. Histology diagnosed 12 MCDA and 44 DCDA twin sets (Fig. 1).

All 5 ultrasound parameters were completed for 36 of the twin pregnancies. An additional 49 pregnancies had incomplete ultrasound information. Each parameter was assessed separately.

Of the cases classified as DC postnatally, the lambda sign was present in 17 of the 30 cases in which it was measured. The lambda sign was not present in any of the MC group. The presence of two placentae was found in 17 of the 44 DC pregnancies, while the remainder had a single placental mass. In the MC group, 10 out of 11 had a single placental mass with one false positive where two placentae were recorded. Discordant sexes were present in only 13 of the 40 DC pregnancies, but in none of the MC pregnancies.

Membrane thickness < 2 mm was found in all but one of the MC pregnancies and, in terms of the number of membrane layers, 2 layers were found in all the MC pregnancies (Table 1).

**Table 1:** Ultrasound findings related to fetal sex, number of placentae, lambda sign, membrane thickness and number of membrane layers compared with histology **Histology**

Ultrasound features	Monochorionic (*n*)	Dichorionic (*n*)	Total (*n*)
Concordant sexes	9	27	36
Discordant sexes	0	13	13
Total	9	40	49
1 placenta	10	27	37
2 placentae	1	17	18
Total	11	44	55
Lambda sign ABSENT	6	7	13
Lambda sign PRESENT	0	17	17
Total	6	24	30
**Membrane thickness**			
< 2 mm	10	10	20
> 2 mm	1	25	26
Total	11	35	46
**Membrane layers**			
2 Layers	9	6	15
3–4 Layers	0	20	20
Total	9	26	35

The correlation between ultrasound features and histology is shown in Table 1.

The correlation between ultrasound parameters and histology when 3 signs were used (sex, placental number and lambda sign) yielded a PPV of 29% and NPV of 97.9%; when an additional 2 signs were added (membrane thickness and number of membranes = 5 signs), the PPV and NPV were 36.3% and 97.9%, respectively.

**Fig. 2:**
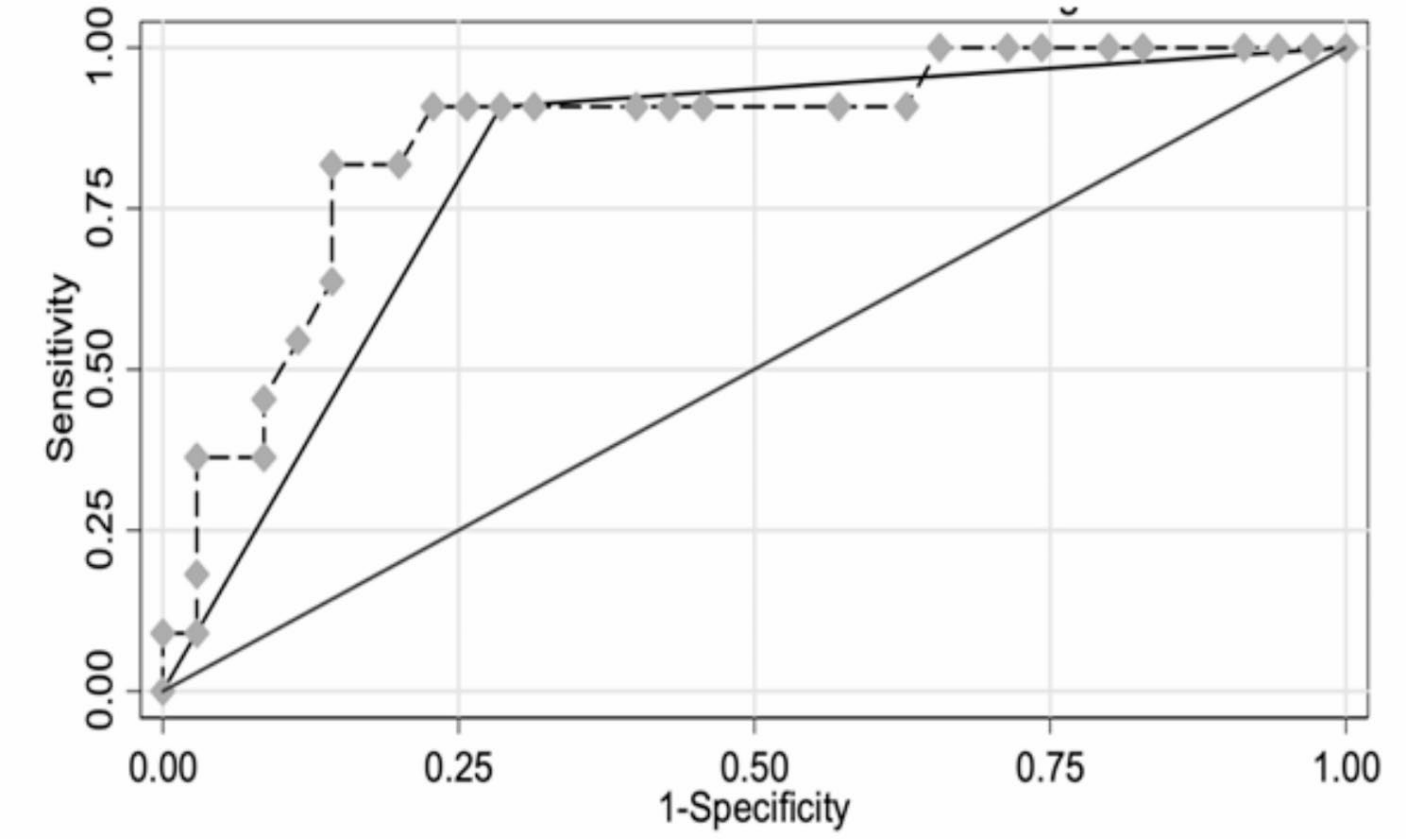
Comparison of ROC curves with and without gestational age ROC, receiver-operated curve; Xb1 = Accuracy of membrane thickness alone Xb2 = Accuracy of membrane thickness taking gestational age into account Difference in area under the curve (Prob > chi2 = 0.1709 - not significant)

Only 8 of the 85 scans were performed in the 3rd trimester, with the majority in the 2nd trimester. However, logistic regression analysis showed that gestational age did not significantly alter the accuracy of membrane thickness as a diagnostic tool (Z-test *p*-value = 0.004; confidence intervals 1.093–5.574; Fig. 2).

## Discussion

We present the findings when three tests for chorionicity are used and evaluate the addition of two further tests. The addition of membrane thickness measurements and the assessment of the number of membrane layers adds only a small amount to the test statistics. Placental number appears to be a poor test to confirm monochorionicity with a very high false positive rate for MC if only one placental mass is seen. The main value of placental number lies in the predictive value of two placentae for dichorionicity. Its overall sensitivity and specificity are low, correlating with the literature which reports a sensitivity of 23% but a specificity of 100% for dichorionicity [21].

The lambda sign remains useful if it is still present beyond 14 weeks. As gestation advances, the lambda sign is likely to disappear as the amniotic sacs expand and compress the membrane take-off area adjacent to the placentae. This data corresponds with existing literature that indicates that the accuracy of the lambda sign decreases with advancing gestational age [13]. A previous study of 410 cases reports a PPV and NPV of the lambda sign after 14 weeks’ gestation of 88% and 94.7%, respectively [13]; however, the present study found a PPV of 46% and a NPV of 100%.

Discordance of fetal sex is very useful to exclude MC twins as discordant sexes are a proxy measurement of dizygosity. MC twins are almost always monozygotic (99,7%) [22]. Therefore, with discordant sexes, we can assume dichorionicity by virtue of the two fetuses being dizygotic.

Concordant fetal sexes may be present in MC or DC pregnancies. The literature on chorionicity does not quote the accuracy of concordant sexes alone to determine chorionicity.

The apparent value of the three diagnostic tests mentioned above lies in their NPV for monochorionicity. These tests appear to be weighted towards diagnosing DC but do not have reciprocal accuracy for diagnosing or excluding MC. For example, discordant fetal sexes are 100% specific for DC, despite concordant sex being only 32.5% specific for MC. The same can be noted for the lambda sign; it can confirm DC pregnancies with 100% sensitivity but its absence can neither confirm nor exclude MC.

Membrane thickness appears to be an accurate and promising test to add to current practice. Importantly, it is not influenced by gestational age, making it a useful tool into the third trimester. If the membrane thickness is < 2 mm, the odds ratio of the pregnancy being MC is 25. If the dividing membrane is > 2 mm, the accuracy is not as high. Previous literature demonstrated an accuracy of 82% for MC [11], whereas this study found a comparable accuracy of 76%. Bracero demonstrated a 96.6% PPV for DC pregnancies with a cut-off of 2 mm in a cohort of 44 pregnancies ranging from 12 to 40 weeks’ gestation (mean 26 weeks) [16]. The difference in results between these two studies can be explained by the small sample size in both (44 and 46 pregnancies).

Townsend, in a cohort of 75 twin sets, reported that the sensitivity for DC drops to 52% in the third trimester (Townsend) [18], whereas this study demonstrated that gestational age (GA) did not decrease the accuracy of membrane thickness measurement. If membrane thickness was modelled against the outcome of histology, a strong correlation was found (*P* > I z I = 0.002).

Counting the number of layers in the dividing membrane proved to be a challenging test to perform as this membrane is only a couple of hundred cells thick. There was a wide margin of intra-observer variation when the layers in the membrane were counted several times. Although the NPV of 4 layers was 100% (95%CI 83.89–100), the specificity was only 77% (95%CI 58–89), giving a PPV for MC of only 60% with a wide confidence interval (36–80). D’Alton found the PPV for DC to be 100% but only 94% for MC [23].

There were 69 consecutive twin pregnancies in D’Alton’s study compared to only 35 in this study. This may explain the discordance of results in the smaller population (MC) (18 in D’Alton’s study vs. 9 in this study).

In formulating a stepwise algorithm, it is important to rank tests and perform them sequentially, starting with tests of high sensitivity but low specificity and gradually moving to tests of higher specificity. In this way, imperfect or less accurate tests can be combined to improve diagnostic accuracy. Determining the correct sequence of these tests is essential to make the diagnosis. The tests in this study all had high sensitivity but disappointing specificity. Table 2 ranks the tests in order of ascending specificity.

**Table 2:** Ultrasound parameters ranked by specificity study findings when 3 signs are used (gender, placental number and lambda sign) and when an additional 2 signs are added (membrane thickness and layers in the dividing membrane = 5 signs in total)

		SENSITIVITY	SPECIFICITY	PPV	NPV
1	Concordance of Sexes	100	32.5	25	100
2	Number of Placentas	90.9	38.6	27	94.4
3	Lambda Sign	100	70.8	46.2	100
4	Membrane Thickness (2 mm)	90.9	71.4	50	96.2
5	Layers in Dividing Membrane	100	76.9	60	100
	3 signs (1,2 and 3)	96.2	43.5	29.1	97.9
	5 signs (1–5 inclusive)	95.7	54.8	36.4	97.9

PPV, positive predictive value; NPV, negative predictive value

If 3 ultrasound parameters are used a PPV of 29.1% and NPV of 97.9% for the diagnosis of monochorionic placentation is calculated

This becomes a PPV of 36.3% and NPV of 97.9% if 5 parameters are used

The tests can also be ranked using Diagnostic Accuracy (D.A.):

$$ {\rm{D}}{\rm{.A}}{\rm{.}}\,{\rm{ = }}\frac{{{\rm{True}}\,{\rm{Positives}}\,{\rm{ + }}\,{\rm{True}}\,{\rm{Negatives}}}}{{{\rm{Total}}\,{\rm{Number}}\,{\rm{in}}\,{\rm{Study}}}} $$

If the tests are performed in sequence of ascending diagnostic accuracy, it may be possible to improve the highest score of accuracy (82.9% for number of layers in the dividing membrane) as the confidence intervals are still large (67.3–91.9) due to limited sample size. Comparison of Tables 2 and 3 shows that the ranking of tests is virtually the same.

**Table 3:** Diagnostic accuracy comparison

	Ultrasound parameter	Diagnostic Accuracy
1	Fetal sex	44.9
2	Number of placentae	49.1
3	Lambda sign	76.7
4	Membrane thickness	76.1
5	Number of membrane layers	82.9

Ranking the lambda sign test in the algorithm was problematic as it had the smallest sample size (*n* = 30) and was not performed if two separate placentae were seen. It has a slightly better diagnostic accuracy than membrane thickness (76.7 versus 76.1) but a wider confidence interval (59.1–88.2 versus 62.1–86.1).

Sample sizes in the existing literature on this topic are generally small and not statistically powered by study population size (*n* = 27–100). [16–18, 23–27] Significance testing has been widely employed to validate these studies with few studies quoting confidence intervals.

Most of the previous literature focuses on a single diagnostic test and none have attempted a statistically-based comparison of cumulative diagnostic ultrasound tests. There are no other studies comparing all five ultrasound parameters as discussed in this study. Classical tests for chorionicity (fetal sex, number of placentae and lambda sign) have a high NPV but are non-specific with extremely poor PPV for MC. Fetal sex and number of placental masses have diagnostic accuracies of only 45% and 49%, respectively.

This study found similar accuracies of membrane thickness and number of layers in the dividing membrane to those that have been reported previously. Some variation in the accuracies can be accounted for by the small sample sizes in all studies in this field. The value of adding these two tests (membrane thickness and number of membrane layers) is that they improve specificity and therefore decrease the number of pregnancies that are erroneously treated as MC. The sensitivities of these tests are 76.1% and 82.9%, respectively.

It may be more appropriate to regard all of these tests as screening tests as we were not able to demonstrate a test with a specificity of greater than 77%. Although employing these tests will still result in an overdiagnosis of MC pregnancies, this number will be reduced. As the rarer, more dangerous condition, this may be considered acceptable.

A strength of the study is that ROC curves, which have not been used in this area of fetal diagnosis before, proved useful to determine cut-off points and evaluate the influence of gestational age.

The limitations of this study are 1) the small sample size was the major limiting factor in interpreting this study.

2) Sonographers did not measure all five ultrasound features in all cases despite this being a prospective study with pre-study education.

**Fig. 3:**
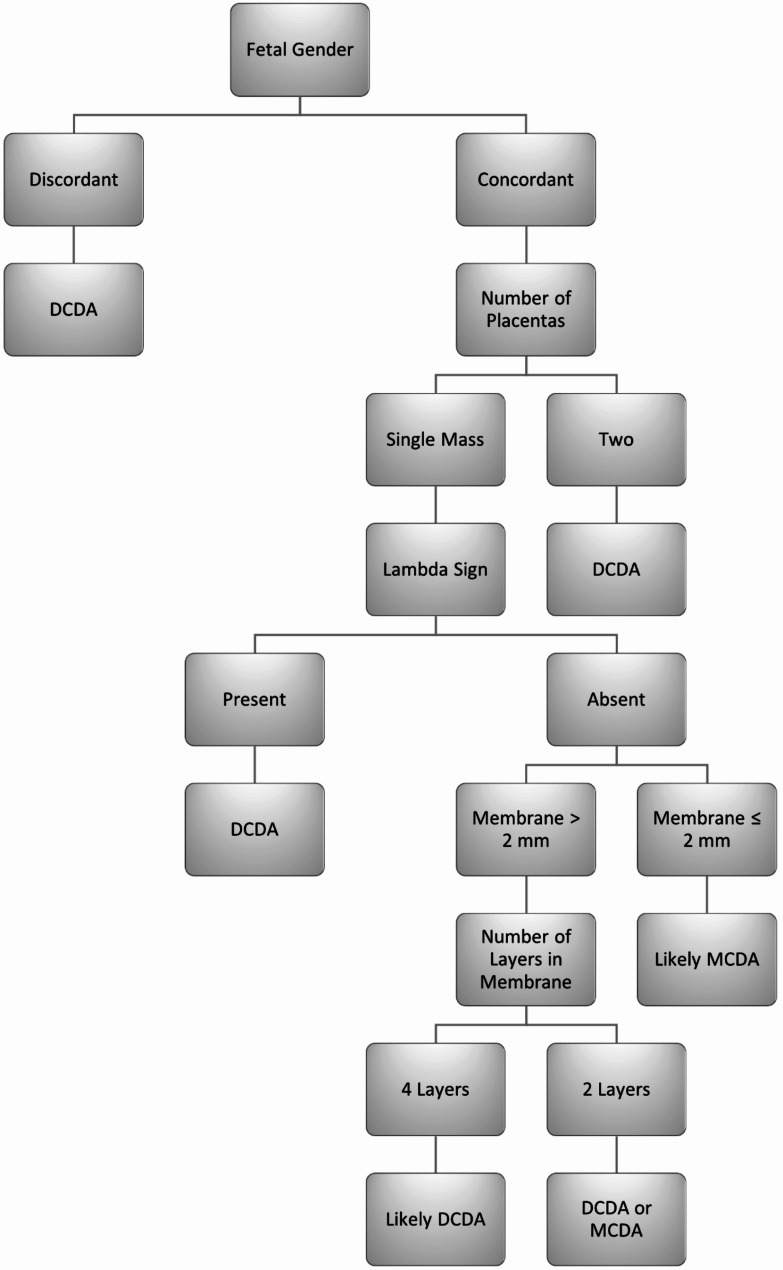
Proposed diagnostic algorithm DCDA, dichorionic diamniotic; MCDA, monochorionic diamniotic

For future research we would recommend measuring membrane thickness in all twin pregnancies with a single placental mass, no lambda sign, and concordant sexes. This will help to decrease the number of pregnancies incorrectly diagnosed as MC. The high NPV (for MC) will ensure that no MC pregnancies are missed. A proposed diagnostic algorithm is described (Fig. 3).

## Conclusions

We described 5 ultrasonographic findings used to predict chorionicity in twin pregnancies who present for care after 14 weeks’ gestation, as is common in developing countries. We found that using 5 parameters rather than the classical 3 provided some improvement in diagnostic accuracy. This study demonstrated that membrane thickness and number of layers remained accurate into the third trimester. This study is also the first to provide statistical evidence for 5 ultrasound parameters to determine chorionicity after the first trimester.

## Data Availability

Data is available from the Dr Zoe Momberg on request.

## Abbreviations

DC: Dichorionic
IUGR: Intrauterine growth restriction
MC: Monochorionic,
MCDA: Monochorionic diamniotic
NPV: Negative predictive value
PPV: Positive predictive value
ROC: Receiver operating characteristic
TRAP: Twin reversed arterial perfusion
TTTS: Twin-twin transfusion syndrome
